# Numerical Simulation and Microstructure Analysis of 30CrMnMoRe High-Strength Steel Welding

**DOI:** 10.3390/ma17174415

**Published:** 2024-09-07

**Authors:** Jimi Fang, Xusheng Qian, Yanke Ci, Cong Li, Xiaoyong Zhang, Kehong Wang

**Affiliations:** 1 School of Digital Technology and Engineering, Ningbo University of Finance and Economics, Ningbo 315175, China; fangjimi@nbufe.edu.cn (J.F.);; 2 School of Materials Science and Engineering, Shanghai Jiao Tong University, Shanghai 200240, China; 3 School of Materials Science and Engineering, Nanjing University of Science and Technology, Nanjing 210094, China; wkh1602@126.com

**Keywords:** numerical simulation, microstructure, high-strength steel, mechanical properties

## Abstract

Welding experiments were conducted under different currents for single-pass butt welding of high-strength steel flat plates. The microstructure of welded joints was characterized using OM, SEM, and EBSD, and the welding process was numerically simulated using a finite element method. According to the grain size obtained by electron microscope characterization and the temperature data obtained by simulation, the microstructure and mechanical properties of coarse grain and fine grain areas of the heat-affected zone were predicted by using the material microstructure and property simulation software. Finally, the results of mechanical properties simulation were verified through mechanical property testing.

## 1. Introduction

30CrMnMoRe high-strength steel has high strength and excellent plastic toughness and is widely used in construction equipment, offshore structures, mining machinery, bridges, building structures, and military mechanical facilities [1,2,3]. With the development of social productivity and demand for energy, the requirements for the load-bearing performance of structural steel plates in fields such as military machinery, large-scale shipbuilding, and offshore drilling platforms are constantly increasing [4,5,6]. Large thickness high-strength steel plates are gradually being applied in these fields. The application of steel plates requires a large number of welded structures, which puts higher requirements on the welding of high-strength steel [7,8,9]. However, there is limited research on the welding of 30CrMnMoRe thick high-strength steel plates.

The welding heat-affected zone (HAZ) of 30CrMnMoRe high-strength steel is prone to martensite transformation, which is not conducive to joint performance [10]. Especially during the welding process of 30CrMnMoRe thick plates, HAZ undergoes multiple thermal cycles, resulting in a complex microstructure and difficult control of mechanical properties [11,12]. Hietala et al. [13] used two different energy inputs to study the fatigue and shear strength of laser-welded lap joints. The influence of multiple weld seam trajectories also has been studied. The test results indicate that there are softening zones in both the heat-affected zone and the weld metal of ultra-high-strength stainless steel welds. When implementing multi-layer and multi-pass welding, each area of the joint undergoes different thermal cycling processes, with the upper layer of the weld and HAZ having a heating effect on the lower layer of the weld and HAZ [14]. Under the influence of these effects, the internal structure of the weld and HAZ will undergo changes, which will affect the performance of the joint [15,16,17]. Bai et al. [18] studied the microstructures and impact toughness of each heat-affected zone (HAZ) formed during rail flash-butt welding. Different thermal simulation cycles were conducted on high-strength carbide-free bainitic rail steel, and the corresponding microstructure was generated using Gleeble-3500 simulator. The results indicate that the peak temperature (PT) affects the transformation products and impact toughness, and the morphology and distribution of martensite austenite (MA) composition largely depend on the welding PT. Scharf-Wildenhain et al. [19] used high-strength steel for arc additive manufacturing, controlling the heat input during the additive process. Welding parameters and AM geometry are correlated with the resulting microstructure, hardness, and residual stress state. High heat input leads to a lower tensile stress in the component and may cause unfavorable microstructure and mechanical properties. The study of the microstructure of different layers in multi-layer and multi-pass welding, as well as the influence of heat transfer between different welding passes on the microstructure, will explain the mechanical properties of multi-layer and multi-pass welding joints from a mechanistic perspective. Conducting research on the evolution of HAZ microstructure in multi-layer and multi-pass welding of 30CrMnMoRe steel, revealing the typical internal microstructure response behavior of HAZ under multiple thermal cycles, is of great significance for regulating the mechanical properties of 30CrMnMoRe multi-layer and multi-pass welding joints.

## 2. Materials and Methods

The base metal used for welding tests in this article is 30CrMnMoRe high-strength steel. The Vickers hardness value range of 30CrMnMoRe quenched and tempered steel is 300–360 HV. In the flat-plate butt welding test, the size of 30CrMnMoRe steel on one side of the flat plate is 100 mm × 50 mm × 5 mm.

When conducting welding tests on high-strength steel, the equivalent residual stress and longitudinal residual stress of the welding specimens obtained using the low strength matching principle are significantly lower than those of the equal strength matching. Therefore, for the welding of 30CrMnMoRe steel, austenitic stainless steel welding wires with lower strength than the base material were selected. The welding wire used for welding experiments in this article is HCr20Ni10Mn7Mo austenitic stainless steel welding wire. After welding with this type of welding wire, the weld seam has a beautiful shape, small and minimal spatter, and has good toughness and crack resistance. This type of welding wire is used for welding high-strength steel and dissimilar steel, such as armor plates, containers, etc. The chemical composition of the base metal and welding wire is shown in Table 1.

**Figure 1:**
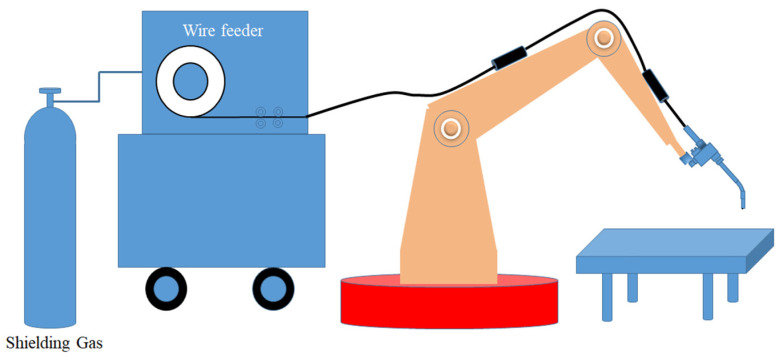
Schematic diagram of welding equipment.

The equipment used for the single-pass welding test of the flat-plate butt joint in this article is the ABB welding robot model IRB2600. The equipment matched with the welding robot includes robot control cabinet, comprehensive power control cabinet, welding power supply, etc., as shown in Figure 1. The single-pass MAG welding was used in this experiment. Three specimens were made for each welding conditions.

The welding parameters for the single-pass welding test of high-strength steel flat plates are shown in Table 2.

The welding specimens were cut, polished, and subjected to corrosion treatment. The corrosion solution was FeCl_3_+HCl+H_2_O. Then, the Olympus inverted metallographic microscope (Olympus Corporation, Tokyo, Japan) was used to observe the microstructure of welded joints. FEI Quanta 250 F (Thermo Fisher Scientific Inc., Waltham MA, USA) field emission environment scanning electron microscopy (SEM) was used to observe the microstructure and tensile fracture of welded joints. The ZEISS EVO MA10 (Carl Zeiss AG, Oberkochen, Germany) electron back-scattered diffraction (EBSD) was used to analyze the microstructure and orientation of the specimen.

**Figure 2:**
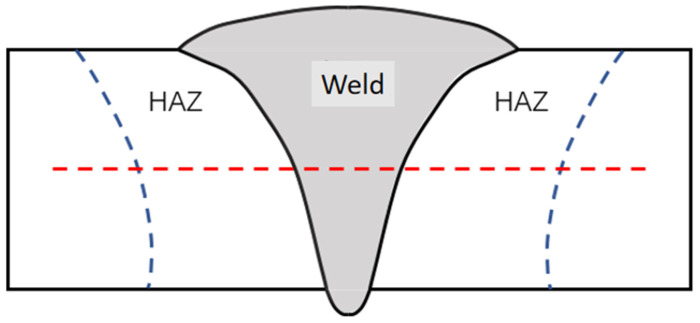
Distribution line of hardness test points. The area between the two blue lines is the heat affected zone, and the red line represents the distribution of hardness testing points.

According to the metal type of the base material and welding wire, micro Vickers hardness was selected as the implementation plan for hardness measurement and the hardness tester model used was HMV-G Vickers hardness tester (Shimadzu Corporation, Kyoto, Japan). When conducting hardness testing on the specimen, the load force was maintained at 5 N for 10 s. The testing direction of the hardness test is shown in the red dashed line in Figure 2.

**Figure 3:**
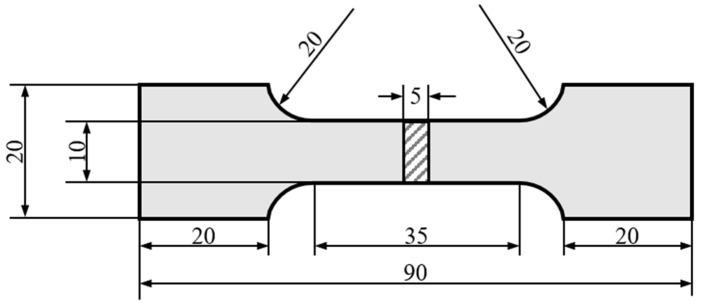
Dimensions of tensile specimens.

The equipment used for tensile testing was the DNS100 universal testing machine (Changchun Institute of Mechanical Science Co., Ltd., Changchun, China), and the specimen size for conducting tensile testing was 90 × 20 × 5 mm. The cutting position of the tensile component and the specimen size are shown in Figure 3.

**Figure 4:**
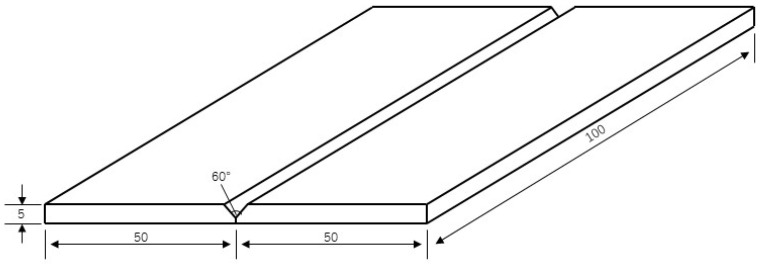
Geometric modeling of high-strength steel single-pass welding.

Using Marc finite element simulation software and combining life and death element technology, finite element simulation of the flat plate welding process was conducted. The geometric modeling of a high-strength steel plate butt joint was established, as shown in Figure 4. The size of the two plates is 100 × 50 × 5 mm, with a 60° groove and a 1.5 mm blunt edge at the bottom. The simulated geometric model is consistent with the actual sample structure dimensions.

**Figure 5:**
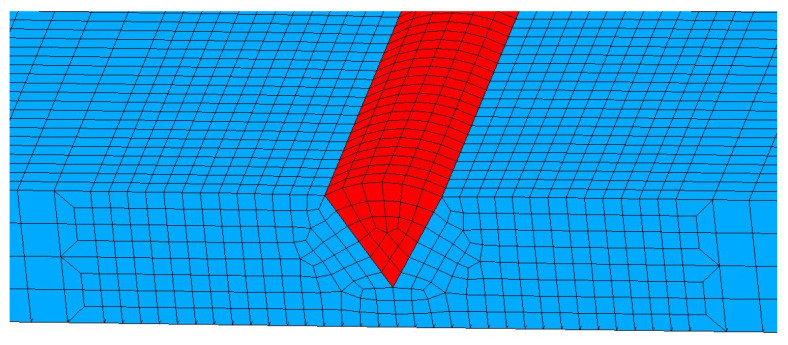
High-strength steel single-pass welding grid model (locally enlarged). The red part is the weld filler metal, and the blue part is the base metal.

After establishing the model, the grid needs to be divided and the local enlarged image of the divided grid model is shown in Figure 5. In order to save computational power, computational speed and computational efficiency are improved and the finite element mesh was optimized by adopting a mesh density transition method when dividing the mesh. In the finite element model used in this section, the total number of nodes is 60,075 and the total number of elements is 46,200. All elements use the eight-node linear heat transfer hexahedral element in MSC. Marc 2020 finite element software.

**Figure 6:**
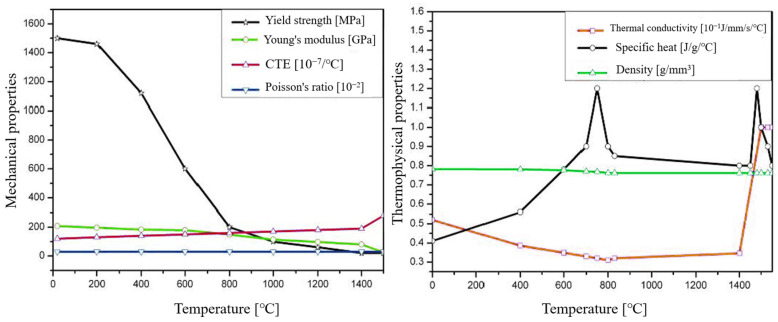
Thermal–physical properties of 30CrMnMoRe steel.

The welding base material is 30CrMnMoRe low-alloy high-strength steel, and the welding wire is HCr20Ni10Mn7Mo austenitic stainless steel welding wire. The thermophysical property parameters of the base metal, including specific heat capacity, thermal conductivity, density, Young’s modulus, Poisson’s ratio, yield strength, etc., are shown in Figure 6. The thermal conductivity of the welding wire was set to 0.215 × 10^−1^ J/(mm · s · °C) and the specific heat to 5 × 10^8^ J/(g · °C), the coefficient of thermal expansion was 1.84 × 10^−5^/K, yield stress was 500 MPa, and other parameters were consistent with the base metal.

**Figure 7:**
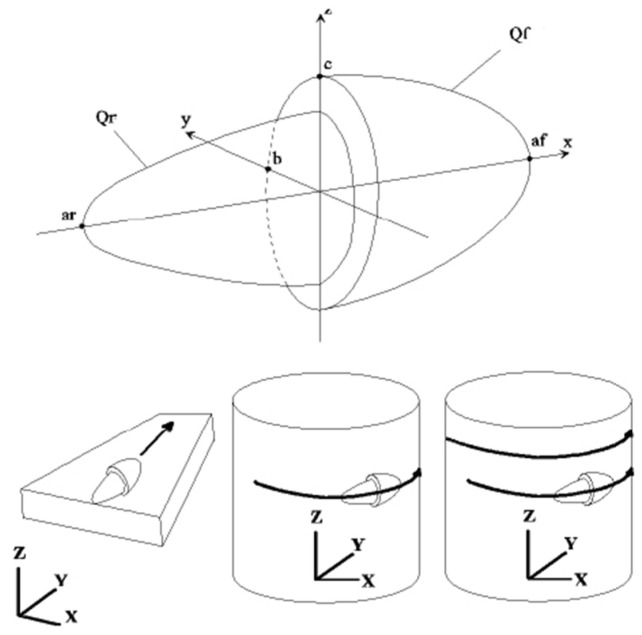
Double-ellipsoidal heat source model. Q_f_: the heat input power of front ellipsoids. Qr: the heat input power of rear ellipsoids. a_f_, a_r_, b, and c are the half axis of the double-ellipsoidal heat source model.

According to the single-pass MAG welding of high-strength steel conducted in this article, the thermal efficiency of the welding heat source is set to 0.7. The distribution of effective thermal power on the specimen can be addressed by using a heat source model. The heat source model used in the finite element simulation in this section is a double-ellipsoidal heat source, with a width of 3.5 mm, a depth of 3 mm, a front end length of 2 mm, and a rear end length of 4 mm. The schematic diagram of the model is shown in Figure 7.

Due to the movement of the welding arc along the welding direction during the welding process, the front and rear directions of the heat source model are not symmetrically distributed, and the heating area on the front side is smaller compared to the rear side. In addition, for a double-ellipsoidal heat source, the models on both the front and rear sides are 1/4 ellipsoids instead of 1/2 ellipsoids. Assuming the welding direction is in the X-axis direction, the power density formula for the front side of the double-ellipsoidal heat source model is:

(1) $$q\left(x,y,z,t\right)=\frac{6\sqrt{3}f_{f}Q}{a_{f}bc\pi \sqrt{\pi }}\cdot exp\left\{-\frac{3{\left[x-\nu \left(\tau -t\right)\right]}^{2}}{a_{f}^{2}}-\frac{3y^{2}}{b^{2}}-\frac{3z^{2}}{c^{2}}\right\},x\geq 0$$

The formula behind the double ellipsoidal heat source model is:

(2) $$q\left(x,y,z,t\right)=\frac{6\sqrt{3}f_{r}Q}{a_{r}bc\pi \sqrt{\pi }}\cdot exp\left\{-\frac{3{\left[x-\nu \left(\tau -t\right)\right]}^{2}}{a_{r}^{2}}-\frac{3y^{2}}{b^{2}}-\frac{3z^{2}}{c^{2}}\right\},x<0$$

In the equation, $a_{f}$, $a_{r}$, b, and c are the half axis of the double-ellipsoidal heat source model; $f_{f}\;and\;f_{r}$ are the heat input ratio of front and rear ellipsoids ($f_{f}+f_{r}=$ 2); and *Q* is the thermal input power.

**Figure 8:**
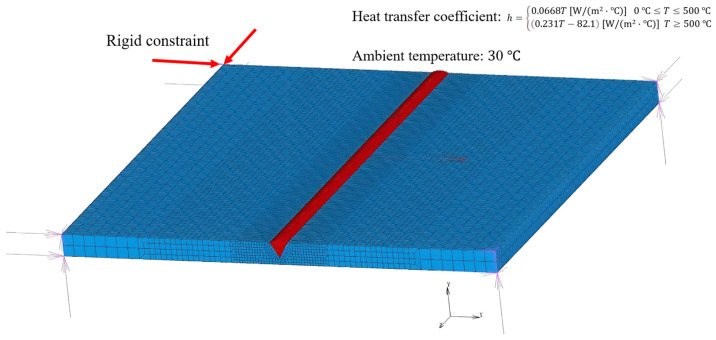
Schematic diagram of displacement constraint application position and boundary condition. The red line is the rigid constraint direction.

For the finite element analysis in this section, the heat dissipation coefficient and the ambient temperature are set as 30 °C. Rigid constraints are assigned to the model to restrict its rigid body movement, as shown in Figure 8. Constraints are applied on the 8 corners of the base plate model, with displacement constraints in the X, Y, and Z directions.

## 3. Results and Discussions

### 3.1. Temperature Field

**Figure 9:**
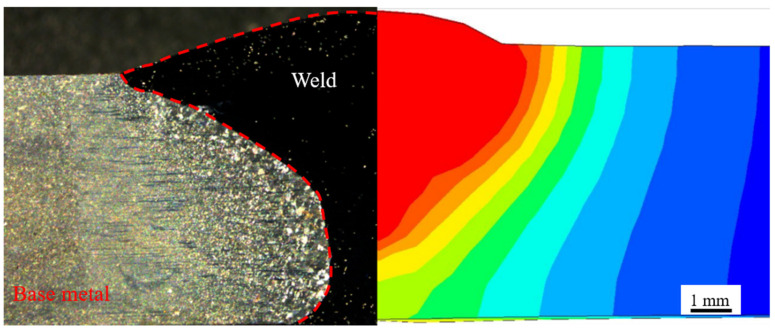
Comparison of actual morphology and simulation results of welded joints.

After applying the conditions, the heat source verification is performed by comparing the cross-sectional morphology of the actual welding specimen with the numerically simulated morphology of the molten pool. The comparison results are shown in Figure 9. The left side of the figure shows the actual joint morphology, and the right side shows the simulation results. From the figure, it can be seen that the morphology of the two molten pools is basically consistent. In actual welding, due to the gap between the two plates when they are docked, there is a connection below the actual molten pool on the left. Overall, the heat source used in this numerical simulation is in line with the actual situation of welded joints.

**Figure 10:**
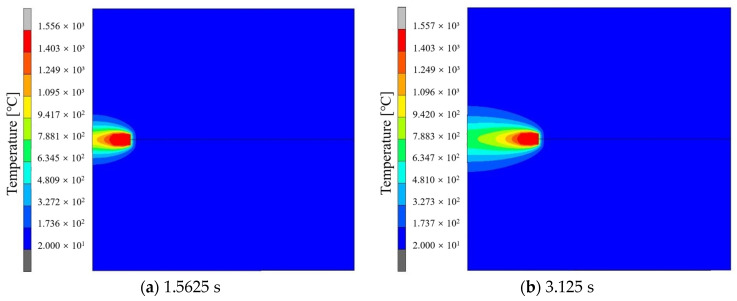

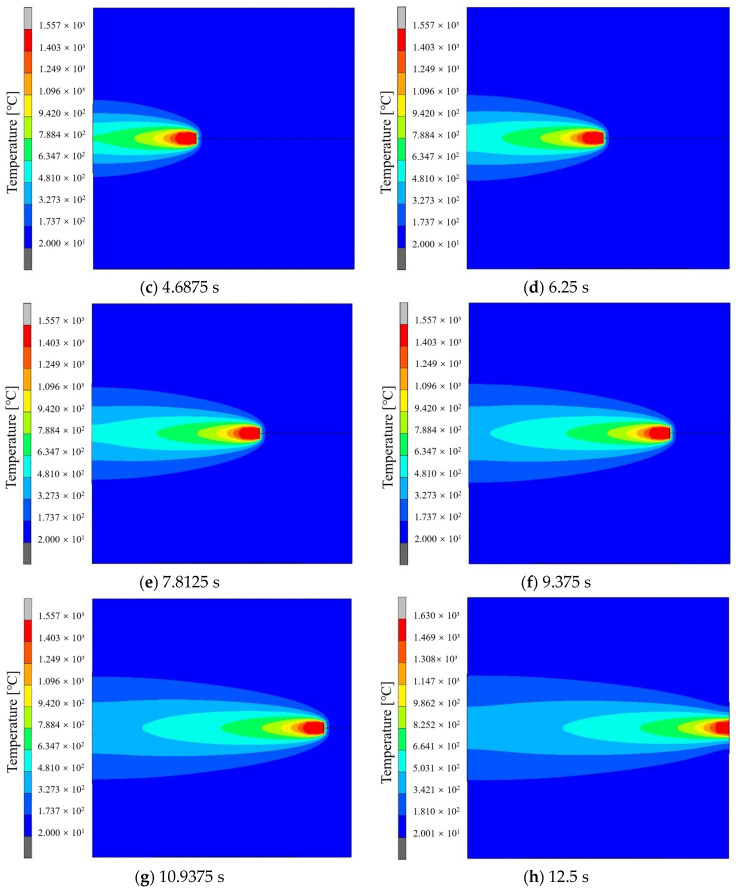
Cloud chart of temperature field at different times.

The transient temperature field cloud diagram of single-pass welding of high-strength steel flat plates is shown in Figure 10. From the figure, it can be seen that, as the welding process progresses, the welding heat source moves and loads along the weld seam, resulting in a dynamic temperature field on the welded part. At the beginning of the welding process, the peak value of the temperature field is slightly lower than that of the subsequent process because the heat flux density loaded through the heat source model has not yet been fully loaded into the welded part. In the actual welding process, after a certain period of arc initiation in the initial stage, the arc gradually reaches a stable state and continues until the end of the welding process. In the simulation results of the welding process, from 3.125 s after the start of welding to 109,375 s at the end, the peak temperature on the welded part remained basically constant, indicating that a relatively stable temperature field had formed on the welded part around 3.125 s after the start of welding, which is consistent with the actual welding process. Unlike the heat from the middle weld seam that can propagate forward in the welding direction, the heat at the end of the weld seam cannot be transmitted forward, resulting in heat accumulation. Therefore, at the end of the welding process, its peak temperature is the highest throughout the entire welding process.

**Figure 11:**
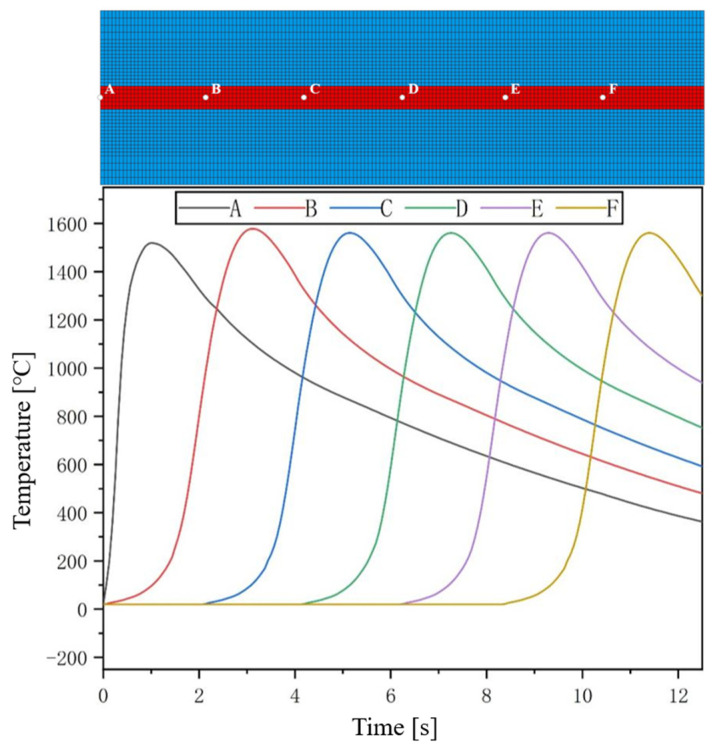
Temperature history curve and point location of the welding direction.

At the center of the weld seam, different points are taken along the welding direction to extract their temperature history curves. The position of each point and the temperature history curve are shown in Figure 11. From the figure, it can be seen that the temperature history curves of each point extracted on the center-line of the weld seam exhibit the same variation pattern, that is, the temperature of each sampling point increases at a high rate with the proximity and arrival of the welding heat source, reaching the peak temperature. Afterwards, as the welding heat source gradually moves away, the temperature of the sampling point gradually decreases. For each sampling point, after welding for 0.5 s, the peak temperature of the point during the entire welding process is reached.

The welding process is an uneven process, specifically a nonuniform rapid heating and cooling process, which inevitably leads to an uneven microstructure and mechanical properties of the welded joint.

**Figure 12:**
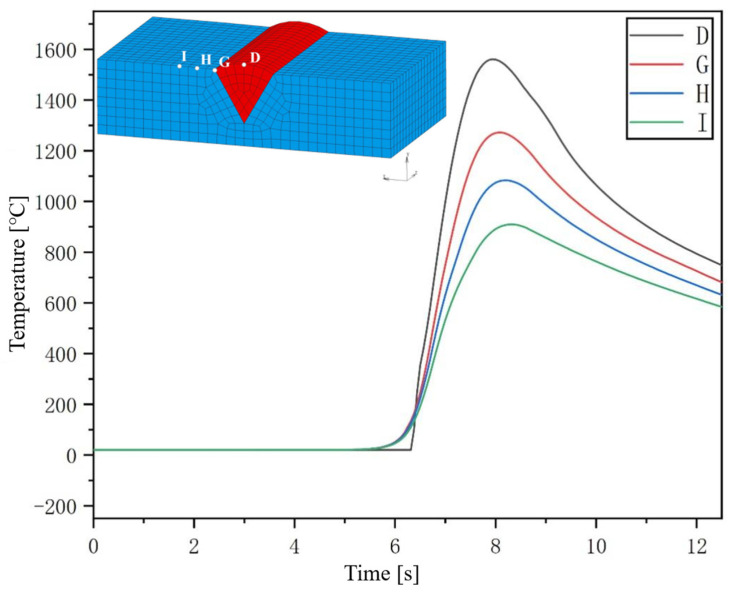
Temperature history curve and point location of the central section.

At the central section of the welded joint, points are taken at four different positions and their temperature history curves extracted. The point positions and temperature history curves are shown in Figure 12. From the figure, it can be seen that, before the welding heat source moves to the central section, the temperature at points G, H, and I on the base metal remains almost unchanged due to the high welding speed relative to the material’s heat conduction velocity. As the welding heat source gradually approaches and ultimately reaches the central position, the temperatures at points G, H, and I are gradually affected by heat transfer and begin to increase. As the welding heat source approaches the central section, the temperature rise rate at the three points also approaches the peak. Due to the direct action of the welding heat source, the heating rate of point A located at the center of the weld seam reached about 1000 °C/s, and the peak temperature reached about 1550 °C. However, austenitic stainless steel does not undergo solid phase transformation during the cooling process after welding, so its final structure is still mainly austenitic with a small amount of ferrite [20,21]. For the joint heat-affected zone, according to the temperature history curves of G and I, the peak temperatures of the coarse grain zone and fine grain zone in the heat-affected zone can be obtained, which are 1280 °C and 900 °C, respectively, reaching the austenitizing temperature. The post-weld cooling rates for the coarse and fine grain regions are 17.5 °C/s and 12.5 °C/s, respectively.

**Figure 13:**
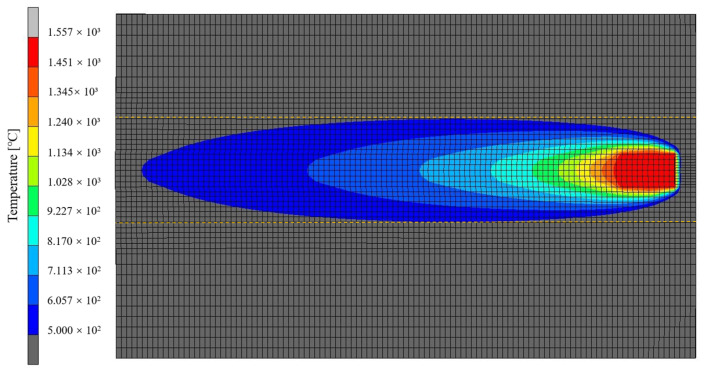
Temperature distribution of HAZ and melting zone. The area between the two yellow interrupted lines is the weld HAZ.

For flat single-pass welding of 30CrMnMoRe high-strength steel, austenitic stainless steel welding wire and high-strength steel base material are different materials. In single-pass welding joints, different nodes undergo different welding thermal cycles due to the influence of welding heat sources, leading to different microstructure evolution processes. This results in changes in the mechanical properties of each node and its final composite in each region of the joint. In Figure 13, the boundaries of weld zone, heat-affected zone, and base metal zone are clearly visible. The orange–red area in the figure is the area with a temperature above 1350 °C. This area is the joint weld zone, and the boundary between the dark blue area is 500 °C. The area between orange–red and dark blue is the HAZ zone. The gray areas on both sides of the yellow dashed line are the base material area. In addition, it can also be seen that the near-weld zone in the heat-affected zone is close to the weld and has a high temperature, so the thermal process it experiences is equivalent to a quenching process. The temperature near the base metal in the heat-affected zone is also higher but lower than that near the weld. The thermal process it experiences is also equivalent to a quenching process. Its peak temperature is higher than the critical temperature and lower than the critical temperature in the coarse grain zone.

### 3.2. Residual Stress and Deformation

**Figure 14:**
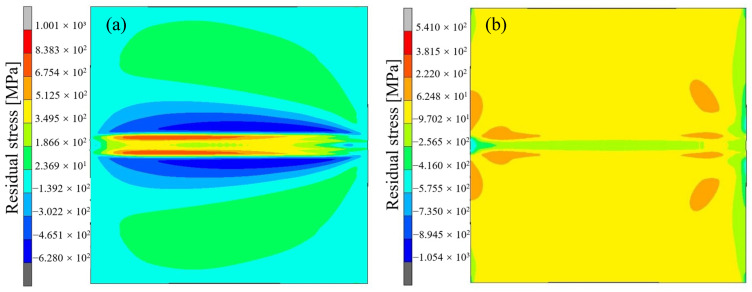
Residual stress contour map at the end of welding. (**a**) Longitudinal residual stress; (**b**) transverse residual stress.

The contour map of residual stress distribution in the welded joint is shown in Figure 14. As shown in the figure, there is a significant tensile stress in the weld seam and heat-affected zone of the welded joint for longitudinal residual stress, especially at the junction between the weld seam and the base metal, where there is a significant stress change. In this boundary zone, there is a peak tensile stress at the heat-affected zone. At a slightly distant location from the weld, it exhibits significant compressive stress, which gradually decreases as the distance from the weld increases. As the distance from the weld seam further increases, some base metal areas exhibit low levels of tensile stress. At the edge of the base metal, it exhibits low levels of compressive stress. For transverse residual stress, there is a low level of compressive stress at the center of the weld seam in the joint, and there is a drastic stress change at the junction of the weld seam and the base metal. Among them, there is a peak tensile stress in the heat-affected zone. As the distance from the weld gradually increases, the transverse stress on the base metal gradually decreases and ultimately presents as a low level of compressive stress.

**Figure 15:**
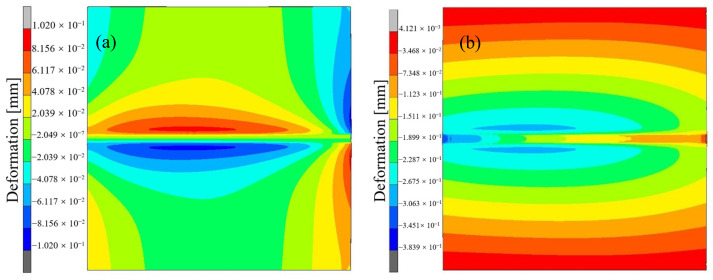
Deformation cloud map at the end of welding. (**a**) X-direction deformation; (**b**) Z-direction deformation.

The displacement cloud diagram of the welded joint in the X direction is shown in Figure 15a. The downward direction is the positive direction of the X axis. Due to the welding direction of the weld seam being the positive direction of the Y axis and the compressive stress on both sides of the weld seam facing the center-line, the opposite deformation situation is shown in the figure. From the figure, it can be seen that there is minimal deformation around the welded specimen due to the displacement constraint applied in the X-axis direction. In addition, the deformation at the beginning of the weld seam is smaller than the deformation at the middle position of the weld seam because subsequent welds will be affected by the squeezing effect caused by the transverse contraction of the previous weld seam, which will generate larger transverse contraction deformation in subsequent welds compared to the previous weld seam. As the welding progresses, the lateral shrinkage deformation gradually increases and stabilizes after reaching a certain level. At the final position of the weld seam, its lateral deformation is relatively small. This is because, at the end of the welding process, there is no metal on the right side of the weld seam at the final position to constrain its thermal expansion process. Only the metal on both sides of the weld seam maintains a certain amount of constraint, so the degree of lateral deformation is relatively light.

The displacement distribution cloud diagram of the welding specimen along the Z-axis direction is shown in Figure 15b. As shown in the figure, due to the displacement constraint in the Z-axis direction applied around the welded specimen before numerical simulation, the deformation along the Z-axis direction at these positions is minimal, almost zero. In addition, due to the influence of lateral shrinkage deformation in the thickness direction of the plate, the angular deformation shows a similar change along the welding direction of the joint weld as the lateral shrinkage deformation, that is, the deformation along the Z-axis direction shows a gradually increasing trend along the welding direction of the weld.

The heat input parameter during the welding process refers to the ratio of linear energy to plate thickness. According to the research content in the references [22,23], when this parameter is less than 10 J/mm^3^, the angular deformation increases with its increase. When this parameter is greater than 10 J/mm^3^, the angular deformation decreases with its increase. The heat input parameters of the single-pass butt welding of high-strength steel flat plates in this study under different welding currents are all greater than 10 J/mm^3^, which is consistent with the literature.

### 3.3. Morphology and Microstructure

**Figure 16:**
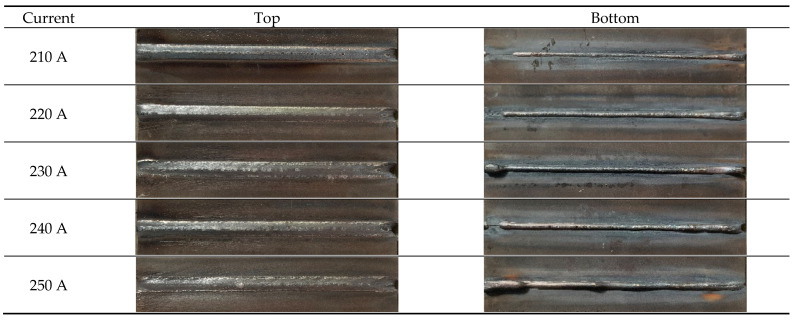
Macro morphology diagram of weld seam.

Figure 16 shows the weld formation obtained using currents of 210 A, 220 A, 230 A, 240 A, and 250 A, respectively. From the figure, it can be seen that, when other parameters are consistent, applying slight changes to the welding parameters (current) results in good weld formation, with smooth and flat front faces and good formability. As the current increases, the higher the heat applied to the weld seam, the higher the penetration of the weld seam. However, under high current conditions, there is a tendency for accumulation at the beginning and end of the weld at the back of the weld. Therefore, it is necessary to control the gap between the two plates more finely during welding to reduce the occurrence of accumulation. Overall, 30CrMnMoRe high-strength steel has good weldability and excellent adaptability to welding processes when MAG welding is used, with a wide welding process window.

**Figure 17:**
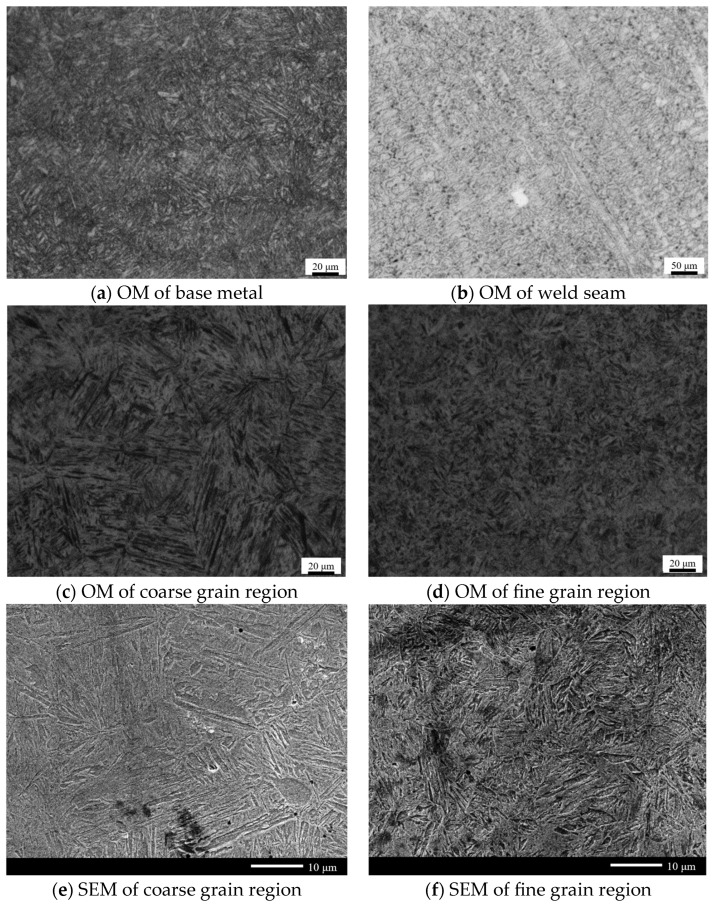
Microstructure morphology of each area of the joint.

Figure 17 shows the typical microstructure morphology of 30CrMnMoRe high-strength steel during single-pass MAG welding. Figure 17a–d show the metallographic structures of the high-strength steel base metal, weld seam, HAZ coarse-grained zone, and HAZ fine-grained zone, respectively. Figure 17e,f show SEM scanning images of the HAZ coarse-grained zone and HAZ fine-grained zone, respectively. The 30CrMnMoRe high-strength steel base material is prepared using quenching and tempering treatment, and its microstructure is mainly tempered sorbite, which has high hardness and strength, as shown in Figure 17a. Figure 17b shows the microstructure of the weld seam, and a large number of dendritic structures can be observed. Due to the use of HCr20Ni10Mn7Mo austenitic stainless steel welding wire, which has a high degree of austenitization, the weld seam structure is basically composed of austenite. The strength, hardness and yield strength of austenite are lower than those of martensite, so the mechanical properties of the weld joint are weaker than that of base metal. This will be verified in the subsequent mechanical property test. Figure 17c,e show the coarse grain structure of the heat-affected zone near the weld joint. It can be seen from the figure that the zone is mainly composed of elongated lath structure and a small amount of granular structure and has a clear austenitic structure outline. It can be seen from the composition of the base metal that the base metal contains a large amount of alloy elements such as Si, Mo, Mn, Cr, etc., which leads to a greater hardening tendency and the formation of martensite structure. In addition, the cooling rate of the coarse grain zone near the crack is slow, which may lead to the production of granular bainite. Therefore, it is speculated that the coarse grain zone is lath martensite, granular bainite, and residual austenite. Figure 17d,f show the microstructure of the fine-grained zone far away from the weld zone in the heat-affected zone. It can be seen from the figure that, similar to the coarse-grained zone, the microstructure in this zone is also lath and granular, accompanied by the contour of residual austenite. During the welding process, this zone is affected by the quenching process, and it is speculated that the fine-grained zone is lath martensite, granular bainite, and residual austenite. In order to further confirm the phase composition in the coarse and fine grain regions, EBSD analysis was conducted subsequently.

**Figure 18:**
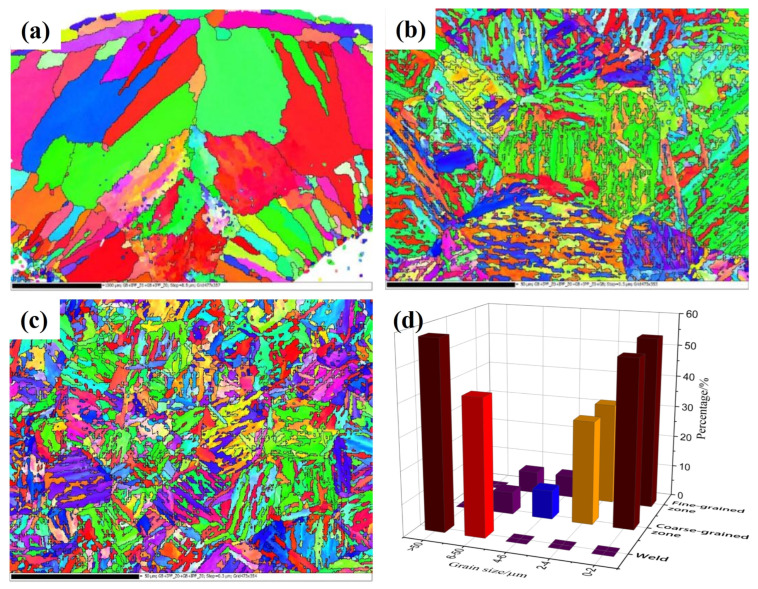
EBSD orientation distribution and grain size in each region. (**a**) Weld zone; (**b**) coarse grain region: (**c**) fine grain region; (**d**) grain size distribution.

Through the metallographic structure of the welded joint, it can be seen that there are differences in the grain size of each area. In order to further explore the distribution law of the grain size and further analyze the influence of microstructure on mechanical properties such as tensile strength, the specimen section is polished by oxide polishing suspension (OPS) and then scanned and observed in different areas of the welded joint through SEM and EBSD. Figure 18 is the EBSD orientation distribution diagram of each area of the single pass MAG welded joint of 30CrMnMoRe high-strength steel. In the orientation distribution map, each region with the same color represents that the region has the same grain orientation. For grains with different colors, the orientation difference is ≥15°. In Figure 18a, it can be clearly seen that the microstructure of the weld seam is mainly austenitic, presenting many large crystal areas with the same orientation, showing the characteristic of columnar austenite grains distributed in the same direction. At the same time, it indicates that, during the welding process, the microstructure in this area undergoes rapid growth due to the influence of heat after austenitization. Compared with the weld zone, the grain orientation in the coarse grain zone of HAZ in Figure 18b is more disordered. It can be seen that there are massive large grains composed of many small grains with similar orientations. The orientation distribution presents typical lath martensite characteristics, indicating that the coarse grain zone is overheated, and the grains grow rapidly after austenitization and then, after cooling, these coarse austenite undergo martensite transformation. The degree of disorder in the grain orientation of the HAZ fine grain area in Figure 18c further increases, similar to the coarse grain area, where there are a large number of blocky large grains composed of many small grains with similar orientations. However, compared to the coarse grain area, the size of the large grains and the small grains that make up the large grains are significantly reduced. In addition, the orientation distribution in the fine-grained region also presents obvious lath martensite characteristics. The coarse grain zone of the heat-affected zone is composed of elongated martensite and granular bainite, and the fine grain zone is composed of fine martensite and granular bainite. The base metal is tempered sorbite, and the hardness of martensite is higher than that of tempered sorbite. Therefore, the hardness values of the coarse grain zone and fine grain zone of the heat-affected zone are higher than that of the base metal, which will be verified in the subsequent mechanical property test.

After calculating the statistical data, the average effective grain size of the weld seam, coarse grain area, and fine grain area is 87.80 μm, 2.87 μm, and 2.36 μm, respectively. It can be seen that the average size of grains in the coarse grain area is 0.5 μm larger than that in the fine grain area. The grain size in the weld seam area is much larger than that in the coarse and fine grain areas. The large-sized austenite grains in the weld zone will make the weld a weak link in the mechanical properties of the joint. Through the heating process during welding, the tempered sorbite in the base metal is completely austenitized, and the austenite grain grows rapidly due to overheating. During subsequent cooling, the grown austenite undergoes martensite and bainite transformation. For the fine-grained region, during the welding process, it is affected by the welding heat source, which is equivalent to undergoing a normalizing process. The grains in the region are refined, resulting in a smaller grain size than the base material. Tempered sorbite in the zone transforms into fine martensite and bainite after austenitizing.

### 3.4. Mechanical Properties

**Figure 19:**
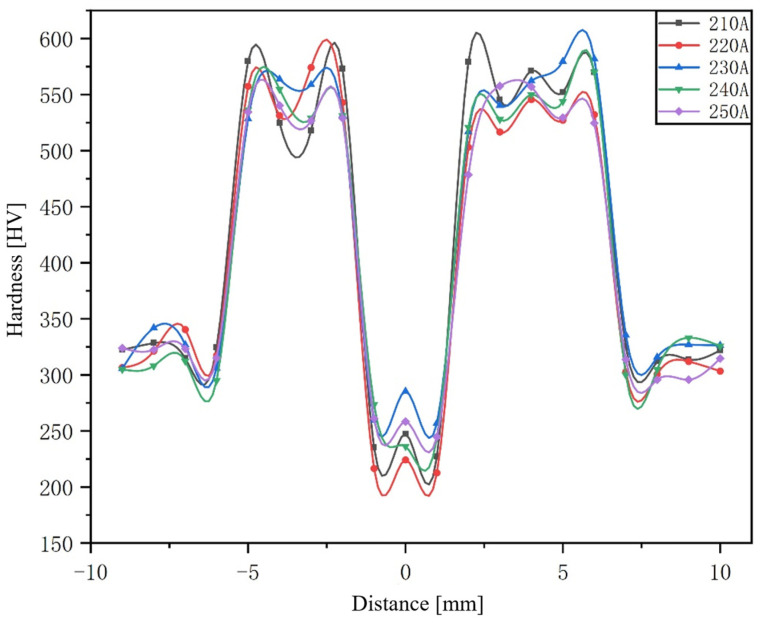
Hardness distribution diagram of central line of joint under different welding currents.

Microhardness testing was conducted on single-pass welded joints of high-strength steel, with the test objects being welded joints obtained under five different welding currents. The hardness test location is shown in Figure 2 and the hardness distribution curve is shown in Figure 19. The average hardness of the base material under different parameters is 330 HV, which is due to the fact that the base material is quenched and tempered steel, with a uniform and fine tempered sorbite structure, resulting in a higher hardness. The hardness of the weld seam is the lowest under different parameters, with an average value of 249 HV. This is because the microstructure of the weld seam area is mainly austenite, and its hardness value significantly decreases compared to the tempered sorbite of the base material. Among these five sets of parameters, the hardness value of the weld seam is the lowest at 220 A and the highest at 230 A.

The hardness value of the coarse grain area is generally high, with an average value of 562 HV. This is because the coarse grain zone is mainly composed of lath martensite. The hardness value of the fine-grained zone is higher than that of the coarse-grained zone, with an average value of 573 HV. The fine grain zone is also mainly composed of flat noodles martensite, whose grain size is smaller than that of the coarse grain zone. In the heat-affected zone, there are a large number of large-angle grain boundaries in the grain boundaries. When adjacent grains with different directions are subjected to force and undergo deformation, in some grains with larger Schmid factor values, the dislocation source leader begins to slip and diffuse along a certain crystal plane and, when the dislocation slides towards the grain boundary position, it is constrained by the grain boundary. In this way, the plastic deformation of grains cannot directly propagate to adjacent grains, which, in turn, leads to the accumulation of dislocations in the plastic deformed grains. The grain size in the fine grain area of the heat-affected zone of the welded joint is finer, and there are more dislocations, resulting in the highest hardness. Among these five sets of parameters, the hardness value of HAZ is relatively low at 220 A and relatively high at 230 A.

The microhardness values in both coarse and fine grain regions of HAZ are much higher than those of the base metal. This is consistent with the judgment of hardness in the foregoing structure analysis, that is, HAZ is mainly composed of martensite and bainite with high hardness, while the base metal is composed of tempered sorbite with relatively low hardness, so the hardness of the base metal is lower than HAZ. The hardness of the single-pass butt welded joint of 30CrMnMoRe high-strength steel flat plate is basically symmetrically distributed in the figure. The weld zone is mainly composed of an austenitic structure and has a lower hardness. The base material is mainly composed of tempered sorbite and has a higher hardness than the weld seam. The heat-affected zone is quenched, and the regional structure is composed of lath martensite, bainite, a small amount of ferrite, and residual austenite, and the hardness is higher than that of the base metal. In the heat-affected zone, because the coarse grain zone is close to the weld, the austenite grain grows rapidly due to the overheating effect of the heat source, and the final recrystallized martensite grain is also relatively coarse. The temperature of the fine grain zone is low, and there is no obvious grain growth. Finally, it is mainly composed of lath martensite with fine grains. The dislocation energy is high, and the hardness is the highest. Five groups of different welding parameters showed similar hardness distribution.

**Figure 20:**
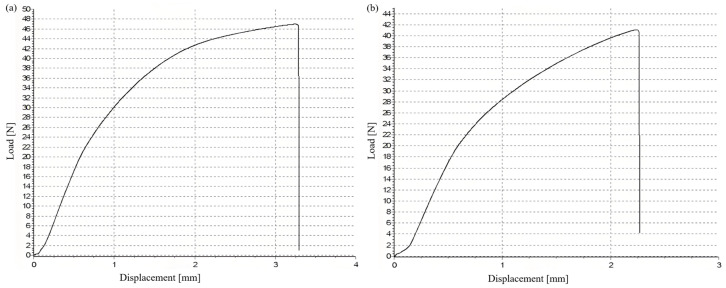

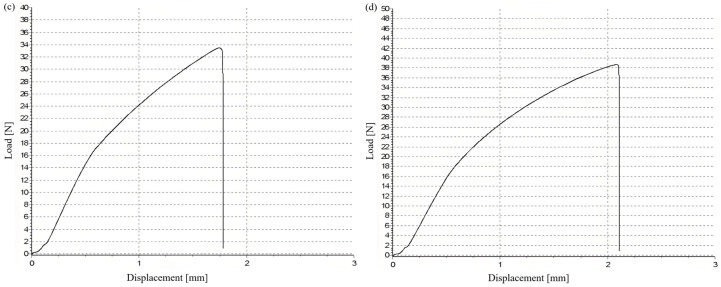
The tensile test curves for the different welding conditions: (**a**) 210 A; (**b**) 220 A; (**c**) 230 A; (**d**) 240 A.

**Figure 21:**
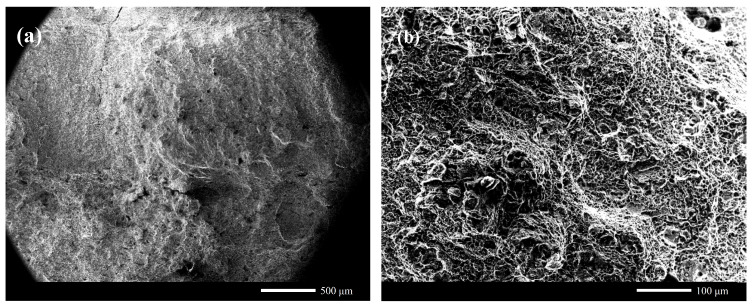
SEM image of joint fracture. (**a**)Macro morphology of fracture surface; (**b**) micro morphology of fracture surface.

Tensile tests were performed on five single-pass welded joints of flat butt joints obtained under different welding currents, and the test results are shown in Table 3. From the table, it can be seen that the yield strength values of the welded joints are all above 350 MPa and the tensile strength values are all above 650 MPa. The tensile test curves for the different welding conditions are shown in Figure 20. The fracture of the joints occurs in the weld seam, and the fracture morphology is shown in Figure 21a. This indicates that the strength of the weld seam is lower than that of the base material, and the weld seam belongs to the weak link in the mechanical properties of the welded joint. As the welding current decreases, both the tensile strength and yield strength of the joint decrease. When the welding current is 210 A, the joint strength is the highest and, when 250 A, the joint strength is the lowest.

The observation of the fracture surface of the 230 A joint after stretching is shown in Figure 21b, with a large number of dimples on the surface, indicating plastic fracture.

## 4. Conclusions

The next step in this field is to study the changes in microstructure under multiple cycles of thermal temperature field and apply it to the wire arc additive manufacturing to solve the stress and deformation problems in the welding process of high-strength steel plates in practical engineering. By controlling the heat input, the microstructure and mechanical properties of the weld and heat-affected zone can be improved.

Based on finite element numerical simulation, the weld seam, near seam zone, and far seam zone underwent different thermal cycling processes, resulting in different microstructure changes. The peak temperatures of the coarse and fine grain zones in the heat-affected zone were 1280 °C and 900 °C, respectively, and the cooling rates after welding were 17.5 °C/s and 12.5 °C/s, respectively.

For microhardness, there is the highest hardness value in the HAZ area of the joint, with an average hardness value of 562 HV in the coarse grain area and 573 HV in the fine grain area. There is the lowest hardness value (lower than the base metal) at the weld seam, with an average hardness value of 249 HV. For tensile testing, the test results show that the yield strength values of the welded joints are all above 350 MPa and the ultimate tensile strength values are all above 650 MPa. The fracture points of the joints obtained under different welding parameters are all welds and plastic fractures.

The microstructure of the weld seam is composed of austenite and a small amount of ferrite, with an average grain size of 87.8 μm. The distribution of grain orientation difference is mainly characterized by large-angle grain boundaries. Through analysis, the microstructure of the coarse grain zone in HAZ is mainly lath martensite, accompanied by a small amount of bainite, ferrite, and retained austenite, with an average grain size of 2.87 μm. The distribution of grain orientation difference is large, and there is a large distribution of small-angle grain boundaries. The microstructure of the fine grain zone in HAZ is also mainly lath martensite, accompanied by a small amount of bainite, ferrite, and retained austenite, with an average grain size of 2.36 μm. The distribution of grain orientation difference is large and there is a large distribution of small-angle grain boundaries.

## Figures and Tables

**Table 1:** Chemical composition of base metal and filler material (wt.%).

Material	Ingredients							
	C	Mn	Si	Cr	Ni	Mo	Re	Fe
30CrMnMoRe (Base Material)	0.26–0.32	1.05–1.55	0.3–0.6	0.8–1.3	-	0.6–0.8	0.07–0.12	Bal.
HCr20Ni10Mn7Mo (Welding wire)	≤0.03	1.00–2.50	0.6–0.9	22.5–24.5	12.0–14.0	-	-	Bal.

**Table 2:** Welding process parameters.

	Single-Pass Welding
Current (A)	210/220/230/240/250
Welding speed (mm/s)	8
Shielding gas	95%Ar + 5%CO_2_
Shielding gas flow rate (L/min)	20

**Table 3:** Tensile test results of the welding joints.

Current (A)	Ultimate Tensile Strength (UTS) [MPa]					Yield Strength (YS) [MPa]					Elongation (A)%
	Average	Max	Min	Median	Standard Deviation	Average	Max	Min	Median	Standard Deviation	
210	889.3	896.1	882.4	889.5	6.8	448.1	457.9	440.1	446.4	9.0	5.5
220	828.5	835.7	824.3	825.5	5.1	436.1	441.5	429.3	437.4	6.2	4.0
230	796.1	802.4	792.8	793.1	4.5	418.2	427.2	411.8	415.7	8.0	4.0
240	771.2	780.3	757.5	775.8	9.9	401.0	405.8	398.2	399.1	4.2	4.0
250	687.1	695.2	675.8	690.3	8.2	385.5	390.7	382.3	383.4	4.6	4.0

## Data Availability

The original contributions presented in the study are included in the article, further inquiries can be directed to the corresponding authors.

## Nomenclature

OM	Optical Microscope
SEM	Scanning Electron Microscope
EBSD	Electron Backscatter Diffraction
HAZ	Heat-Affected Zone
PT	Peak Temperature
MAG	Metal Active Gas Arc Welding
OPS	Oxide Polishing Suspension
UTS	Ultimate Tensile Strength
YS	Yield strength
f_f_	Heat input ratio of front ellipsoids
f_r_	Heat input ratio of rear ellipsoids
Q	Thermal input power
